# Developing guided self-help for depression using the Medical Research Council complex interventions framework: a description of the modelling phase and results of an exploratory randomised controlled trial

**DOI:** 10.1186/1471-244X-8-91

**Published:** 2008-11-24

**Authors:** Karina Lovell, Peter Bower, David Richards, Michael Barkham, Bonnie Sibbald, Chris Roberts, Linda Davies, Anne Rogers, Judith Gellatly, Sue Hennessy

**Affiliations:** 1 School of Nursing, Midwifery & Social Work, The University of Manchester, Manchester, UK; 2 National Primary Care Research & Development Centre, The University of Manchester, Manchester, UK; 3 School of Psychology, University of Exeter, Exeter, UK; 4 Department of Psychology, The University of Sheffield, Sheffield, UK; 5 School of Community Based Medicine, The University of Manchester, Manchester, UK; 6 Department of Health Sciences, University of York, York, UK

## Abstract

**Background:**

Current guidelines for the management of depression suggest the use of guided self-help for patients with mild to moderate disorders. However, there is little consensus concerning the optimal form and delivery of this intervention. To develop acceptable and effective interventions, a phased process has been proposed, using a modelling phase to examine and develop an intervention prior to preliminary testing in an exploratory trial. This paper (a) describes the modelling phase used to develop a guided self-help intervention for depression in primary care and (b) reports data from an exploratory randomised trial of the intervention.

**Methods:**

A guided self-help intervention was developed following a modelling phase which involved a systematic review, meta synthesis and a consensus process. The intervention was then tested in an exploratory randomised controlled trial by examining (a) fidelity using analysis of taped guided self-help sessions (b) acceptability to patients and professionals through qualitative interviews (c) effectiveness through estimation of the intervention effect size.

**Results:**

Fifty eight patients were recruited to the exploratory trial. Seven professionals and nine patients were interviewed, and 22 tapes of sessions analysed for fidelity. Generally, fidelity to the intervention protocol was high, and the professionals delivered the majority of the specific components (with the exception of the use of feedback). Acceptability to both professionals and patients was also high. The effect size of the intervention on outcomes was small, and in line with previous analyses showing the modest effect of guided self-help in primary care. However, the sample size was small and confidence intervals around the effectiveness estimate were wide.

**Conclusion:**

The general principles of the modelling phase adopted in this study are designed to draw on a range of evidence, potentially providing an intervention that is evidence-based, patient-centred and acceptable to professionals. However, the pilot outcome data did not suggest that the intervention developed was particularly effective. The advantages and disadvantages of the general methods used in the modelling phase are discussed, and possible reasons for the failure to demonstrate a larger effect in this particular case are outlined.

## Background

Depression is a significant cause of personal distress, social disability and economic consequences for patients, families and wider society [1]. Cognitive behaviour therapy (CBT) is a crucial treatment for depression [2], but access to CBT is characterised by long waiting lists [3]. The adoption of a 'stepped care' system has been proposed to overcome problems of access [4]. Stepped care seeks to enhance the effectiveness of service delivery by providing low intensity 'minimal interventions' to a proportion of patients in the first instance. These interventions are generally described under the broad label of 'self-help' where CBT techniques are used by a patient and facilitated through a health technology such as written materials or computer programmes. In the United Kingdom, guidelines for depression recommend the use of 'guided self-help' at step 2, between 'watchful waiting' and brief psychological therapy [2]. Guided self-help is defined as involving a CBT-based self-help resource and limited support from a health care professional. However, there remains ambiguity concerning the best way to deliver guided self-help, such as the most appropriate 'health technology' for the delivery of the self-help materials (written materials or multimedia), the level and nature of the guidance required, and the skills and expertise required to deliver this guidance.

**Figure 1:**
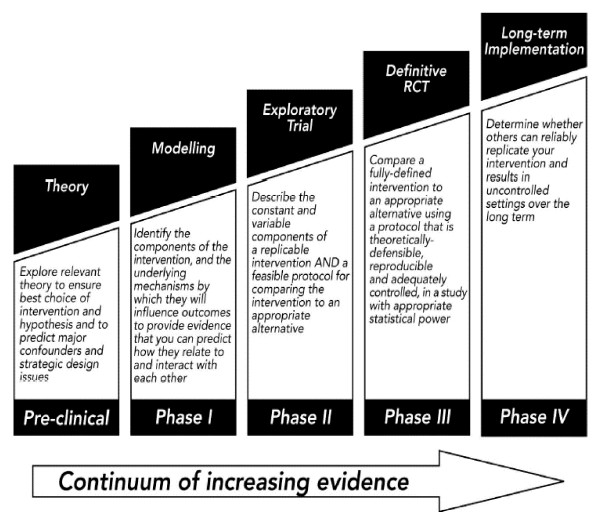
MRC Framework for Complex Interventions.

Where there is ambiguity about an intervention and the best way to deliver it, the Medical Research Council (MRC) recommends a phased development process [5]. Phase 1 involves 'modelling', which requires theoretical and empirical work to 'identify the components of the intervention and the underlying mechanisms by which they will influence outcomes' (Figure 1). Phase 2 then involves an exploratory RCT to test the intervention, examine delivery in routine settings, and provide estimates of key trial parameters such as recruitment rates and estimates of effectiveness, prior to a definitive trial.

There is still significant ambiguity about the optimal way to conduct the modelling phase. For example, the MRC suggests the use of 'computer simulations, or economic modeling qualitative testing through focus groups, preliminary surveys, case studies, or small observational studies.' Whatever approach is used, the challenge is to help develop interventions that are 'evidence based' (i.e. based on current best evidence about patient outcomes) and patient-centred (i.e. in line with the preferences and needs of patients)[6]. The utility of methods used for the modelling phase will also be judged by their resource implications and efficiency in delivering information to assist in intervention development in a timely manner.

We have developed a process for the modelling phase which may have utility for future intervention development work. This paper presents this model and describes its application to the development of a guided self-help intervention.

## Methods

**Figure 2:**
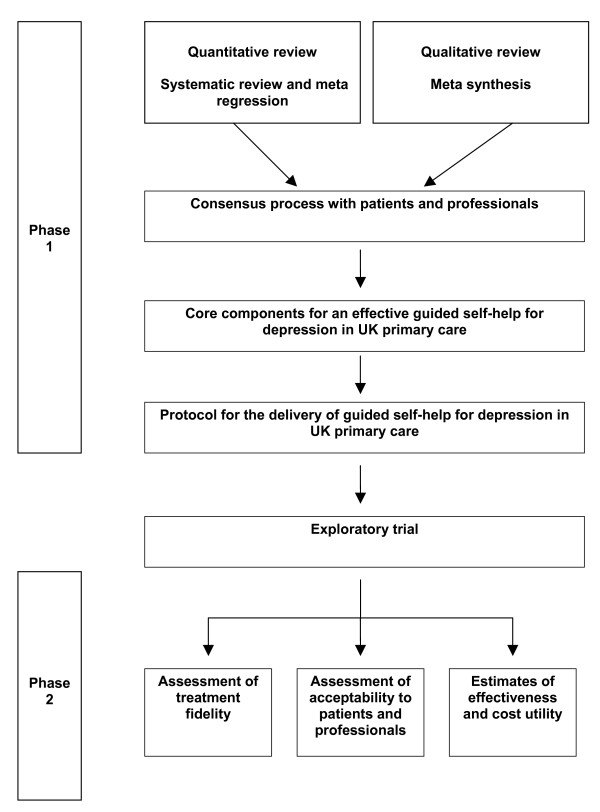
Structure of the intervention development process.

The process of the study is shown in Figure 2 and involved two phases. Phase 1 involved modelling using secondary research and a consensus process to develop a protocol for the delivery of guided self-help. Phase 2 involved an exploratory randomised controlled trial to test fidelity, acceptability and effectiveness of the protocol.

### Phase 1 Modelling

Phase 1 involved quantitative (meta regression) and qualitative (meta synthesis) reviews to synthesise available evidence on the effectiveness of the intervention and identify key factors that may moderate effectiveness [7,8]. The results of these previously published reviews are summarised in Table 1. Evidence from these reviews was important for modelling the intervention, but the reviews were not sufficient, as they raised new questions, and were unable to deliver specific answers on important clinical and service delivery issues. Therefore, the results of the reviews were combined with a consensus process [9,10] which sought to interpret the evidence and deal with the ambiguities that remained (the consensus process is detailed in Tables 2 and 3).

**Table 1:** Methods and results of the meta regression and meta synthesis

**Systematic review and meta regression **[7]

Previous reviews and electronic database searches were used to identify relevant studies. For inclusion, studies had to be randomised controlled trials with populations suffering from depression or depressive symptoms. The intervention had to assist patients in the treatment of their symptoms, using a health technology such as written information, audiotape, videotape or computer presentation. Interventions were designed to be conducted predominantly independent of professionals (3 hours or less). Data on moderators of treatment effect (i.e. the patient populations, study design, internal validity, and intervention content) and outcomes were analysed using meta regression.

Thirty four studies were identified with 39 relevant comparisons. Results found that the overall effect of self-help interventions was 'medium' according to the current convention, with the pooled standardised mean difference -0.43, 95% CI -0.57 to -0.30. The variation in effect size attributable to heterogeneity (I2) was 77.3%. When the analysis was restricted to studies using 'guided self-help' the pooled standardised mean difference was large (-0.80, 95% CI -1.01 to -0.58), and the variation in effect size attributable to heterogeneity was 68.3%. Effectiveness of guided self-help was related to population factors such as recruitment in non-clinical settings, and recruitment of patients with existing depression rather than those 'at risk'. Aspects of the intervention which moderated effects included contact with a therapist, and the use of CBT techniques. In the subset of 'guided self-help' studies using therapist contact, there were no significant associations between outcomes and the number of sessions, their content, delivery mode or the background of the therapist.

**Meta-synthesis **[8]

Qualitative work is ideally suited to capture the complexity of care processes, and as such has a key role to play in the development of complex interventions. This study used ***meta synthesis***, which has some similarity to quantitative meta-analysis, involving the development of an overview of research, but based on qualitative papers.

The meta-synthesis involved 1) Identifying the literature and appraising the studies. 2) Data analysis and interpretation, including extraction of main findings, synthesis of main findings into an explanatory framework, and application of the explanatory framework to the guided self-help intervention.

Medline, Embase, Cinahl, and Web of Knowledge were searched from 2000 – 2005. The British Sociological Association criteria for the evaluation of qualitative research papers was adapted to appraise the studies. The researchers looked across the different papers for common and recurring concepts.

From the 9 papers included in the review 5 key themes were determined: personal experience of depression, ambivalent help seeking and the covert presentation of psychological problems, control and helplessness in engaging with treatment, stigma associated with treatment and patients' understanding of self-help interventions. This broad explanatory framework was then applied to the specific issue of the development of the guided self help intervention.

**Table 2:** Methods and results of the consensus process

*Phase 1 – Consensus exercise*

The results of the studies summarised in Table 1 were used as evidence to develop the intervention. Despite the reviews, significant ambiguities remained that could not be answered with the review evidence. To make decisions concerning these areas, we conducted a consensus exercise.

We identified a total of 32 experts/key stakeholders including international academics, mental health professionals and service users with knowledge/experience of self-help techniques for depression. Potential participants were sent an invitation to take part detailing the rationale for the exercise, a summary of the results from both the meta regression and meta synthesis and a copy of a consensus questionnaire. Those who did not respond within 4 weeks were sent a follow-up invitation. Limitations in funding and time meant that a single questionnaire was used, and feedback of responses and a second questionnaire were not used.

The content of the questions is shown in Table 3. Questions related to the number, duration and time period of the intervention, how to incorporate and manage issues such as the patient being the agent of change and regaining control, the delivery mode of the guidance, the health technology, and the training and role of health professional delivering the intervention.

Nineteen individuals (59%) responded. Eight were academics, 10 were health professionals (4 GPs, 3 psychologists, 1 psychiatrist, 1 nurse, 1 primary care mental health worker). One respondent was a service user. Consensus was present in the following areas:

1) The importance of patient preference for the delivery mode of the intervention (i.e. telephone, email or face to face delivery)

2) The provision of materials in alternative formats such as a CD for those with literacy or concentration difficulties.

3) The inclusion of information on recognition and relapse strategies

4) The importance of highlighting the role of the patient as the agent of change

5) Although differences occurred in terms of number, duration and spread of sessions, the ranges of these were relatively limited.

6) Although there was agreement that definitions of depression and consideration of prior coping strategies should be incorporated into the intervention, there were some concerns that endorsing patients' prior views and short term coping strategies could be disadvantageous, and suggested the need for the facilitator to ensure that such coping strategies were helpful.

**Table 3:** Content of the consensus questionnaire

1. Given the evidence (or lack or it) could you indicate the maximum and minimum number of guided self-help sessions you feel would be appropriate?

2. Given the lack of evidence could you indicate maximum/minimum session duration?

3. Could you indicate the time period the sessions should be delivered over?

4. What methods could we use to ensure that patients are familiarised to the treatment model, to communicate that they are expected to be the principal agent of change?

5. To what extent should we build in choices (face to face, telephone, email) in terms of how the guidance is provided to patients?

6. How can facilitators and materials reconcile the tension for patients between regaining control over their emotional wellbeing whilst accepting the need for help?

7. Should we ensure that the facilitation and materials in the self-help process include a theme of remoralisation (experience of improvement not the end point)? If so, how?

8. In the self-help process, to what extent should we explore the causal origins of a person's depression as opposed to its maintenance?

9. Are there factors which you think impact the development and maintenance of a therapeutic relationship within a guided self-help model? If so, which ones?

10. Whilst we cannot provide computer delivered materials, to what extent should we produce the material in a range of alternative media?

11. In choosing the self-help material we have determined that the material must be CBT based. However we are less certain about whether the material should also have the following attributes and would welcome your views.

How important are the following factors in your opinion

a. Material reflects patients' lay definitions of mental health problems
b. Material reflects patients' previous coping strategies (e.g. distraction)
c. Material contains information on recognition and relapse strategies
d. Material contains information on pharmacological interventions
e. Material contains information of aspects of living with depression that are not explicitly addressed by the intervention e.g. stigma, material support
f. Material contains information on a return to social functioning rather than symptom relief

12. Are there any other attributes that you believe are essential, if so which ones?
13. What specific interpersonal competencies should the facilitator possess in order to develop a therapeutic relationship/alliance with the patient?

14. What specific therapeutic competencies should the facilitator possess in order to engage the patient to 'self manage?'

15. Should we assume existing training (professional or other) leads to these facilitator (both interpersonal and therapeutic) core competencies? If yes, what type of and level of core training should facilitators have already undergone to select them? If no, what education and training should be provided?

16. What group of health-care workers, if any, would be best suited to deliver guided self-help?

To combine the results of the meta regression, meta synthesis and consensus process, we drew up a matrix of the results, with each column of the matrix detailing one of the 'core components' of the intervention that we wished to address, and the rows referring to the results from each of the three data sets (meta regression, meta synthesis and consensus process).

The matrix used in the current study is shown in Table 4. This matrix was used as the platform for a discussion within the trial team to derive the final intervention to test in the Phase 2 exploratory trial. The rows labelled 'Incorporated into the intervention' indicate the final decision about the nature of the intervention along the 'core components'. A full description of the final intervention is provided in Table 5.

**Table 4:** Matrix used for synthesis of findings concerning the 'core components' of GSH

	**Level of guidance**	**Nature of guidance**	**Health Technology**
**Meta regression findings**	Number of sessions not related to outcome	Better outcomes where guidance provided, and where based on CBT	No differences between technologies (email, telephone, face to face)

**Meta synthesis findings**	No relevant findings	No relevant findings	No relevant findings

**Consensus exercise**	Agreement on timing, duration, and number of sessions	Agreement that patient preference should determine the nature of guidance	Agreement that health technology should be accessible, and help with literacy problems.

**Incorporated into the intervention**	3–10 sessions, 15–30 minutes duration over 5–12 weeks	CBT based. Patient preference delivery of guidance	Devised a self-help manual and also a CD

	**Who should deliver guided self-help**	**Personal experience of depression**	**Ambivalent help seeking and covert presentation of problems**

**Meta regression findings**	No differences in outcome between professional and paraprofessionals	No relevant findings	No relevant findings

**Meta synthesis findings**	No relevant findings	Personal experience characterised by feeling of inability to cope, and disturbances to functioning. Use of lay language/metaphors important	Point in illness trajectory where people make service contact, and their prior contact with other help may determine acceptability

**Consensus exercise**	Most frequent were nurses and primary care graduate workers. Specific training needed	Mixed response to inclusion of lay language and metaphors. Agreement on importance of social functioning, and relapse prevention	None relevant

**Incorporated into the intervention**	Primary care graduate workers or other mental health professionals	Emphasised return of social functioning. Lay language, metaphors and causal explanations included. Relapse prevention incorporated	Expectations and prior contact emphasised and included in the intervention. Choices and patient preference for interventions included

	**Control and helplessness in engaging with treatment**	**Stigma associated with treatment**	**Patients' understanding of self-help**

**Meta regression findings**	No relevant findings	No relevant findings	No relevant findings

**Meta synthesis findings**	Patients reported coping strategies such as distraction, or the use of locations associated with feelings of safety and control	Extent to which guided self-help acknowledges issues of stigma likely to determine acceptability	Seeing the self as the agent of change may be very important

**Consensus exercise**	Mixed response but emphasis on collaborative working, patient centred goals, and roles i.e. patient as change agent	None relevant	Agreement of collaborative working, explicitly detail roles of both patient and MHW i.e. patient as change agent, coach as facilitator

**Incorporated into the intervention**	Highlighting intervention as a method of regaining control and incorporating use of coping strategies termed 'respite' in the intervention	Discussed guided self-help as requiring a sense of acting on the world and enhancing self-worth	Explicit team rationale, with the patient as 'team captain', facilitator renamed as 'self-help coach'

**Table 5:** Content of the Recovery programme guided self-help intervention

The key components of the intervention included a book [13] and guidance from a health professional. The intervention consisted of an evidence based health technology guided by a mental health professional (termed 'self-help coaches'), delivered over 3–10 sessions, 15–30 minutes per session over a period of 5–12 weeks. The book entitled the 'Recovery Programme for Depression' (also recorded onto CD-ROM) was divided into 4 steps. Details of the steps and their content can be found below.

The recovery book was written to engage patients, and incorporated metaphors, lay language and personal experience. The book was printed using colour, illustrations, and each step was colour coded for easy reference, and had a Flesch readability ease score of 74.0. The book, CD, and printed diaries were all placed in a plain black folder in an attempt to ensure a level of privacy for patients.

Mental health workers ('self-help coaches') attended a two day training programme which ran on both study site settings. The training focussed on all aspects of delivering the intervention from initial assessment, delivering the rationale for treatment and guiding patients with the materials. Training was accompanied by a training handbook (available from the authors). A significant portion of the training was spent practising self-help coach skills and working through the 4 steps of the book (using fictitious but typical cases of mild to moderate depression).

Step 1 'What is this recovery programme all about' introduced guided self help, emphasising the pivotal role of the patient as the agent of change and in control of their intervention. We highlighted this by stating that the recovery programme was about a 'team', with the patient as the 'team captain'. In addition we renamed the mental health workers (MHW) 'self – help coaches' and used the analogy of a personal fitness trainer to further highlight the view that the coaches were there to support, monitor and advise patients as opposed to the traditional therapist role. Case vignettes were used to demonstrate the personal experience of depression.

Step 2 'Understanding the way I feel' incorporated the notion that people's experience of depression is focussed on their inability to cope and loss of social functioning. This was addressed by suggesting that patients complete an 'Impact sheet' to highlight areas of loss or reduced functioning. We gave examples of typical metaphors people use to describe depression in lay language. The ABC model of emotion (feeling, thinking, and doing) was used to assist understanding of depression. Brief written exercises were included to assist engagement and understanding. To further ensure that the patient was in control of their treatment a section was included on devising patient centred goals, which were outcomes that the patient wanted to achieve.

Step 3 'My recovery programme' focussed on 3 evidence based interventions which were principally CBT based and included the rationale and application of behavioural activation, cognitive restructuring and ways to improve physical problems such as sleeping, irritability and concentration. These interventions were highlighted as a method of regaining control and thereby improving functioning. Patients were asked to choose the intervention that they thought would best help them. To assist this choice and ensure patient preference, patients were asked to read the 3 recovery stories at the end of the book. These stories were typical but fictional cases demonstrating people's experience of depression, guided self-help and recovering from depression using one of the 3 interventions.

Step 4 'Staying well and the recovery stories' focussed on advice and ideas on continuing to manage mood and relapse prevention. The recovery stories were fictitious (though based on clinical experience) accounts of people experiencing depression and managing depression using one of three interventions i.e. behavioural activation, life style changes or cognitive restructuring.

### Phase 2

Having completed Phase 1 modelling, Phase 2 involved an exploratory randomised controlled trial to test the intervention developed in Phase 1. This paper reports empirical data on three key aspects of the Phase 2 exploratory trial:

1. Examination of fidelity using analysis of taped guided self-help sessions. Fidelity refers to the extent to which delivery of the intervention is consistent with the treatment protocol and was delivered 'as planned' [11].

2. Examination of acceptability to patients and professionals through qualitative interviews. Identifying acceptability of a complex intervention is a critical precursor to a definitive trial [5] and is informed by emerging theory about the successful implementation of complex interventions in routine clinical settings, which suggests that the extent to which new innovations in health settings become 'normalised' depends on whether they are able to be routinely embedded in everyday clinical practice [12].

3. Preliminary estimation of the intervention effect size through comparison of outcomes in patients receiving the intervention and those in a 'usual care' control group.

### Phase 2 – Fidelity

We aimed to examine fidelity to the guided self-help protocol. Data on use of the manual [13] and session uptake was recorded. A rating manual was developed based on the manual devised for those providing the intervention (so called 'self-help coaches') which defined specific tasks to be carried out in session 1 and in sessions 2 to 10 (the manual is available from the authors). The components extracted for the fidelity study are shown in Table 6.

**Table 6:** Components of the treatment to be rated for adherence

Session 1 components and subcomponents (SC)

(1) Orientate client to the session
(2) Explicitly state 'team' approach (3 SCs)
(3) State roles (2 SCs)
(4) Initiate patient centred interview with impact sheet (11 SCs)
(5) Complete PHQ (2 SCs)
(6) Introduce book (4 SCs)
(7) Educate briefly about depression
(8) Write down ABCs
(9) Write down personal links
(10) Encourage patient to complete step 2
(11) Encourage patient to read recovery story in step 3
(12) Seek feedback on session
(13) Clarify if there are further questions
(14) Agree next appointment.

Sessions 2–10

(1) Review depression
(2) Review risk
(3) Review progress on the intervention
(4) Collaboratively plan next stage of intervention
(5) Collaboratively plan new intervention
(6) Seek feedback on intervention
(7) Ask final questions
(8) Agree next appointment.

In addition, session 2 contained:

(1) Review helpfulness/completion of tasks in book
(2) Review goals
(3) Review what the patient thinks about stories
(4) Relate stories to interventions
(5) Encourage patient to choose intervention.

The data pool comprised a total of 36 audio-taped sessions out of a possible 78 sessions of guided self-help representing 46% of delivered sessions and 7 of the 10 'self-help coaches' involved in the exploratory trial. The dataset was restricted because of an uneven distribution of tapes across sessions for the self-help coaches (e.g., tapes for sessions 8 onwards represented a single self-help coach with the same patient). Accordingly we devised a pragmatic design which assured a minimum of 1 tape being sampled from session 1 and 1 tape from sessions 2–10 for each of the 7 self-help coaches. In total, 22 tapes (i.e. 61% sampling density) were independently rated by 2 masters-level graduates with prior experience of analysing language who had received training on the fidelity manual together with reading the source material (i.e. self-help manual). The two raters received tapes in random order to protect against systematic biases. They were instructed to listen to each tape in the specified order and code whether each major component and subcomponents were present. The use of two independent raters enabled a kappa statistic (*k*) to be calculated summarising the agreement regarding the presence or not of the required techniques and procedures.

### Phase 2 – Acceptability

Permission to undertake qualitative interviews was granted by York Ethics Committee. Patients and professionals were approached and asked to consent to an interview. Semi structured interviews took place either on the telephone or face to face. Two interviewers were used. One interviewer had a psychology background with some clinical experience (JG), while the other was a researcher with a social policy background (SH). Patients were asked questions about pre-trial experience of depression ('Did you try and manage depression before seeking help from the GP'), the process of treatment ('How did your guided self-help sessions take place and what did you think about the format and frequency of these sessions?', 'Have you used or looked at the book or listened to the CD at all or not') and outcome ('How are you feeling now, compared with when you first saw your GP about feeling low?'). Questions for professionals included those about the intervention ('In what ways do you think guided self-help represents a different or similar way of dealing with mental health problems to the way in which you approached your work with people with depression previously?'), the patients they treated ('How do you feel the recovery programme worked for each of the clients?', 'What did you think of the suitability of these clients for guided self-help?') and clinical supervision ('Do you have any comments about the supervision that you received from the trial clinicians?').

All interviews were audiotaped and transcribed verbatim. Data were analysed using a framework analysis by KL and SH [14]. An initial coding framework was developed and transcripts were checked against the framework to ensure that there were no significant omissions. Codes in each interview were examined across individual transcripts as well as across the entire data set and allocated to the framework. Using aspects of the constant comparative method of analysis [15] broader categories used linking codes across interviews. Data were interpreted and analysed within the framework to distil, interpret and structure component statements about the intervention.

### Phase 2 Effectiveness

Permission to undertake the exploratory randomised controlled trial was granted by York Ethics Committee. Patients were recruited in the north of England from GP practices and referrals to primary care mental health teams. Inclusion criteria were age 17 and over, with mild to moderate depression, scoring 14–28 on the Beck Depression Inventory II (BDI-II) [16], and consenting to participate. Patients were excluded if they were in current psychological treatment for depression (e.g. brief psychological therapy), had suicidal intent, post-natal depression, bereavement reaction, or primary drug or alcohol dependence. Current antidepressant use was not an exclusion criterion. Due to recruitment difficulties early in the trial, the inclusion criterion on the BDI-II was eventually increased to an upper limit of 45.

The guided self-help intervention was delivered according to the protocol detailed earlier. Clinical supervision for the self-help coaches was provided by KL and DR on a two weekly basis, and was delivered via phone or face to face depending on clinician preference and work availability. The control intervention was treatment as usual from the patient's GP.

The primary outcome was symptoms of depression as measured by the BDI-II [16], a 21 item self report scale (each item rated 0–3) which produces a depression severity score ranging from 0–63. The scale is designed to correspond to criteria for the assessment of symptoms relating to a diagnosis of depressive disorder [17].

Several secondary outcomes were included. The CORE-OM [18] is a 34-item measure of psychological distress and comprises four dimensions: subjective well-being, symptoms, functioning, and risk. For the purposes of the current study, the mean score on the 34 items (each item rated 0 to 4) was used. Following recommended procedures, scores were multiplied by 10 for ease of interpretation, and thus range from 0 to 40. The PHQ-9 [19] scale is a measure of depression severity which has 9 items corresponding to DSM-IV depression criteria (each item rated 0–3), with scores ranging from 0 to 27. Both scales perform well against a diagnostic gold standard in primary care [20].

The Social Adjustment Scale SAS-SR [21] is a measure of social function. The 45-item scale (each item rated 1 to 5) has subscales measuring respondents' functioning in relation to paid work, housework, social and leisure activities, and relationships with close and extended family. Respondents completed all relevant subscales, but for the purposes of the analysis a average score was calculated across all completed items, with scores ranging from 1 to 5.

Economic outcomes were also collected and will be reported separately. All assessments were completed at baseline and three months post-randomization by trained assessors.

A conventional power calculation was inappropriate for this exploratory trial. Instead, to assist with decision making about a later definitive trial, 50 patients per arm were deemed sufficient to give a reasonable indication of the likely effect size. Assuming attrition of 20%, the trial sought to recruit 125 patients.

Patients were individually randomised to guided self-help or treatment as usual. Allocation was minimised by age, gender and severity of depression. Initially severity was limited to BDI-II scores of 14–28 and the minimisation strata were scores of 14 – 19 (mild) and 20–28 (moderate) [16]. Following recruitment difficulties and the decision to include higher levels of severity in the trial, a further stratum was added (29–45) for severe depression (the BDI-II manual suggests scores of 29–63 are severe, but the cut-off of 45 was used because very severe cases were considered to be inappropriate for guided self-help). To ensure concealment of allocation, the researcher making judgements of eligibility contacted a central randomisation service by telephone to receive the allocation.

Data were analysed on an intention to treat basis, with patients analysed in their allocated groups regardless of attendance for treatment. The main analyses used analysis of covariance in Stata, controlling for baseline scores of each outcome variable (BDI II, CORE-OM, PHQ9 and SAS) and the minimisation variables (BDI-II, age and sex). Missing outcome data were not imputed. Following the decision to change the BDI-II severity inclusion criteria for the trial from 14–28 to 14–45, a subgroup analysis was conducted to examine whether the treatment effect was moderated by initial severity [22]. The analysis of the primary outcome was re-run including an additional variable representing the interaction between initial severity (14–28, and 29–45) and group allocation.

## Results

### Phase 2 recruitment

**Figure 3:**
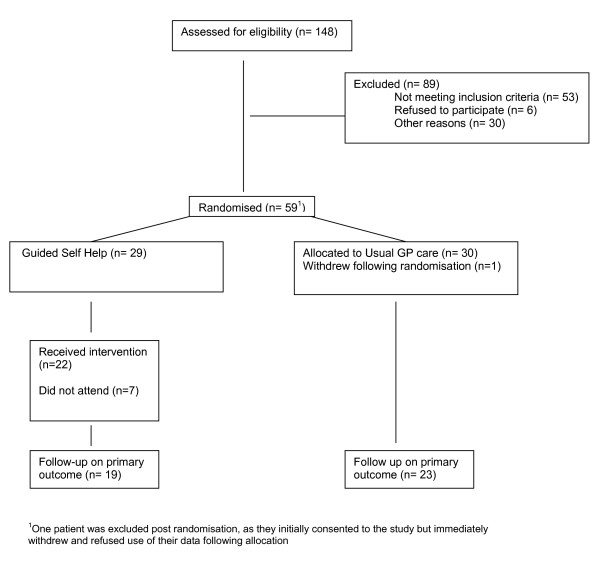
Consort diagram.

A total of 58 eligible patients were recruited to the trial (46% of those initially planned) and 42 (72%) provided data on the primary outcome at 3 months. Patient flow is shown in Figure 3. The socio-demographic characteristics of the study participants are summarised in Table 7. Seventy four per cent of the sample were female, mean age 37.6, and 93% described their ethnic origin as white. Only 28% were in full time employment and 24% had no educational qualifications. The majority (69%) were taking antidepressant medication.

**Table 7:** Socio demographic characteristics of the patients included in the trial

	**Guided self-help** **n = 29**	**Treatment as usual** **(n = 29)**	**Total** **n = 58**
**Sex**			
male	9 (31%)	6 (20.7%)	15 (25.9%)
female	20 (69%)	23 (79.3%)	43 (74.1%)
**Age**			
Mean (sd) range	35.3 (10.8) 19–60	39.9 (13.6) 20–63	37.6 (12.4) 19–63
**Ethnicity**			
White	27 (93.1%)	27 (93.1%)	54 (93.1%)
Black African	1 (3.4%)	2 (6.9%)	3 (5.2%)
Black other	1 (3.4%)	0	1 (1.7%)
**Marital Status**			
Single/unmarried	14 (48.3%)	10 (34.5%)	24 (41.4%)
Married/co-habiting	11 (37.9%)	14 (48.3%)	25 (43.1%)
Divorced/separated	4 (13.8%)	3 (10.3%)	7 (12.1%)
Widowed	0	2 (6.9%)	2 (3.4%)
**Living Situation**			
Alone (with or without children)	11 (37.9%)	12 (41.4%)	23 (39.7%)
With husband/wife	6 (20.7%)	11 (37.9%)	17 (29.3%)
With partner	7 (24.1%)	4 (13.8%)	11 (19.0%)
With parents	4 (13.8%)	1 (3.4%)	5 (8.6%)
With other relatives	1 (3.4%)	0	1 (1.7%)
Other	0	1 (3.4%)	1(1.7%)
**Qualifications**			
Degree or Equivalent	6 (20.7%)	6 (20.7%)	12 (20.7%)
Higher Educational qualification	8 (27.6%)	4 (13.8%)	12(20.7%)
A-Level or equivalent	1 (3.4%)	3 (10.3%)	4 (6.9%)
GCSE grade	6 (20.7%)	9 (31.0%)	15 (25.9%)
No formal qualifications	7 (24.1%)	7 (24.1%)	14 (24.1%)
Other	1 (3.4%)	0	1 (1.7%)
**Employment**			
Full-time	8 (27.6%)	8 (27.6%)	16 (27.6%)
Part-time	7 (24.1%)	3 (10.3%)	10 (17.2%)
Self-employed	2 (6.9%)	1 (3.4%)	3 (5.2%)
Voluntary employment	1 (3.4%)	1(3.4%)	2 (3.4%)
Sheltered employment	0	0	0
Unemployed	7 (24.1%)	6 (20.7%)	13 (22.4%)
Student	2 (6.9%)	4 (13.8%)	6 (10.3%)
Housewife/husband	2(6.9%)	4 (13.8%)	6(10.3%)
Retired	0	1 (3.4%)	1 (1.7%)
Other	0	1 (3.4%)	1(1.7%)
**Currently taking antidepressants**			
Yes	23 (79.3%)	17 (58.6%)	40 (69.0%)
No	6 (20.7%)	12 (41.4%)	18 (31.0%)
**Baseline measures (mean SD n)**			
BDI (II)	27.97 (8.4)	29.97 (8.1)	28.97 (8.3)
CORE-OM	18.89 (5.38)	18.74 (5.54)	18.81 (5.42)
SAS	2.64 (0.6)	2.82 (0.4)	2.73 (0.6)
PHQ9	14.96 (5.7)	14.79 (5.1)	14.87 (5.5)

### Phase 2 – Fidelity

Of the 29 allocated to the guided self-help intervention, 22 (76%) attended at least 1 session. The mean number of sessions attended was 3.5 (SD 3.5, range 1–10). Mean duration of session 1 was 53.5 minutes (SD 8.6) and mean total duration of the entire treatment was 132.3 minutes (SD 97.6). Most sessions were delivered face to face with only 8 sessions delivered by telephone and 1 by email.

The overall pairwise kappa agreement for session 1 was 0.79 (n = 7 cases) and for sessions 3 and 4 was 0.73 (n = 5 cases). There were insufficient tapes to yield kappas for other sessions. Overall, pairwise agreements for individual components ranged from unity (i.e., *k = *1.0) for 14 components to a single occurrence of 0.0 for just one component (feedback). For session 1 data, 9/14 components obtained unity agreement (*k *= 1.0), one component yielded *k *= 0.77, three others ranged between 0.36–0.50, and a single component yielded a value of 0.0. For sessions 3 onwards, 5 out of 8 components obtained unity agreement and the other three components yielded *k *values between 0.15–0.36. As an indicator of the extent to which self-help coaches worked according to the protocol, an average of 10 out of 13 components were utilised (range 8–12) for each of the sessions.

### Phase 2 – Acceptability

Eight of the 10 self-help coaches were interviewed (Table 8) and 9 patients undertook post-treatment qualitative interviews (Table 9). The main themes emerging from the professional and patient interviews are described below.

**Table 8:** Characteristics of the professionals (self-help coaches) for the qualitative study of acceptability

**ID**	**Role**	**Gender**	**Years of experience**	**Professional qualification**	**Prior use of guided self-help?**
01	Graduate primary care mental health worker	F	2.5	Postgraduate certificate in mental health	Yes
02	Mental Health Lead	F	16	Registered mental nurse	Yes
03	Graduate primary care mental health worker	M	3	Postgraduate certificate in mental health	Yes
04	Mental health link worker	F	21	Diploma in Occupational Health	No
05	Mental health link worker	F	23	Qualified social worker	No
06	Graduate primary care mental health worker	F	1	Postgraduate certificate in mental health	Yes
07	Graduate primary care mental health worker	F	1	Postgraduate certificate in mental health	Yes
08	Graduate primary care mental health worker	F	25	Registered mental nurse	Yes

**Table 9:** Characteristics of patients interviewed for the qualitative study of acceptability

**ID**	**Gender**	**Age**	**Pre BDI score**	**Post BDI Score**	**Sessions received**
01	F	52	32	23	3
02	M	34	28	20	1
03	F	47	22	5	3
04	F	44	25	12	10
05	F	55	24	5	9
06	F	59	31	22	5
07	F	36	21	0	10
08	F	48	20	12	9
09	F	48	20	27	4

BDI – Beck Depression Inventory

### Professional perceptions of guided self-help compared to existing ways of working

Guided self-help was not an unfamiliar concept, and all were able to recognise similarities between it and their previous way of working. The differences lay in the codification and systematic treatment of specific elements such as advice. The 'Recovery Programme' book was described as being focussed and enabled the patient to make more of an informed choice about the interventions.

The format of being able to give the patient this information in one booklet and giving them more information to make an informed choice of what path they chose to take is different to how we would work. (Self-help coach 07)

### Professional perceptions of guided self-help

In terms of the overall self-help package (the book, CD and diaries) the self-help coaches welcomed its presentation, quality and the use of colour.

It was an all in one pack, it wasn't sort of bits....I think in self-help, we might use odd diaries that are photocopied from other places and it's sort of, bits of scraps of here and there – So it was much more, you know, I guess, in the spirit of what guided self-help is... that the patient guides themselves. (Self-help coach 03)

Self-help coaches found the CD acceptable, and recognised the usefulness of this delivery mode, but felt that it could be more engaging. For most coaches the use of the telephone to deliver the intervention was a departure from their usual practice, but was welcomed as a means of increasing access to patients. Recovery stories were highlighted as particularly useful.

I think the recovery stories are a good way of explaining to somebody what they'll actually be doing in an intervention. I think 'cos obviously we still have the problems of any psychological intervention is a bit mysterious for patients who haven't experienced it before. So having those stories was a really good way of like showing them what would be expected of them. (Self-help coach 6)

### Professional perceptions of suitability, severity and outcome for individual patients

Self-help coaches agreed that patients in the study were similar in terms of severity, problem presentation, and outcome to the patients seen in routine clinical practice. Self-help coaches reported difficulties engaging some patients who had complex problems or severe depression, whilst they reported other patients with severe depression as showing considerable improvement. It was also felt that some problems were more amenable to counselling, particularly relationship problems.

*Patient has done well... doing well – huge benefits from the intervention- client expressed problem with sleep- she identified goals – similar to other people I see. (Self-help coach 8, Patient pre BDI 20 post 12)*.

*Patient more difficult to engage.... more severe than my other patient- more significant issues – complex. (Self-help coach 7, Patient pre BDI 40 post BDI 27)*.

*worked well with this client- doing BA- very suitable for GSH- even though her score was high. Yes, yeah. I think even though, perhaps, her score at the beginning ... we would considered them originally too high. (Self-help coach 7, Patient pre BDI 28, post BDI 0)*.

*Patient was difficult to engage (even though depression was mild and perhaps more appropriate for counselling) (Self-help coach 1, Patient pre BDI 14, post BDI 15)*.

### Professional perceptions of barriers to incorporating guided self-help into routine clinical work

Self-help coaches felt that barriers to using guided self-help in practice included patients' prior expectations of mental health services, the severity or complexity of patients' problems, and barriers raised by the health professionals themselves. For example some coaches felt that patients expected counselling, and thus a self-help model would not match such expectations, whilst others acknowledged that they themselves had difficulty using a self-help approach because they had been trained in other psychological models and found the change difficult. Self-help coaches (particularly those that previously worked with patients for hour long sessions) found the reduced time with clients challenging. A further barrier to incorporating guided self-help into usual clinical practice was a lack of self-help materials (either in terms of content or presentation) and it was evident that the materials used in the trial were seen as high quality, which clinicians felt was important.

The patients often aren't familiar with guided self-help; they're more familiar with the counselling approach. So it can be difficult at first and there can be a bit of, bit friction trying to get the patient into the, the self-help framework, rather than a kind of counselling session. (Self-help coach 7)

*If you've worked in other ways I think it's really quite difficult. As a new worker being taught only this model then yes it makes a lot of sense. But I think when, when you're coming from, you know, as many years of having worked with other models. Even if you've worked in a variety of models, again there's the time issue that's been really quite difficult I think to, to just cut down what you do, so much. (Self-help coach 2)*.

I think, sometimes the lack of good quality resources does get in the way. I think as I mentioned earlier, like clinically we give out sort of bits of paper and things and it's actually nice having presented, you know, a whole program that you can hand over to someone and say "this is what we're going to be working on". I enjoyed actually being able to give something, you know, highly presentable. (Self-help coach 3)

### Patients views of guided self-help compared with previous treatment

All but one patient had previous episodes of depression. Some had received no treatment, others had been prescribed medication or seen a health professional. Both positive and negative views were expressed about previous mental health care, and most patients made favourable comparisons of guided self-help with previous mental health care.

Last time I almost felt that I'd come out from the counsellor and I'd sort of blurted out all the stuff I need to but then it didn't seem to get me anywhere. I mean you can tell the stories over and over again, and you can feel justified in feeling hurt, upset or whatever about a situation but it doesn't change it. So the Big Ear doesn't really do it for me. (Patient 4)

I felt it was an hour where it was just me and her talking and it was getting stuff off my chest really, which at the time was quite helpful but I think for me the guided self-help came at the right time because that felt a lot more practical, the guided self-help felt more sort of practical building blocks for getting my life back on track whereas the counsellor was just venting steam really. (Patient 9)

### Patients' views of guided self-help materials and delivery

Patients characterised guided self-help as helping take back control of their lives; being empowering; providing choices of different approaches to take; and being practical. One patient felt that guided self-help was too simplistic. Most patients viewed the material favourably, particularly the recovery stores.

I really thought the booklet was well organised and very interesting as well and nicely written. It was kind of well written in a simple way, without being patronising or condescending. (Patient 5)

### Patients' views of the role of guidance and the self-help coach

Patients' described their self-help coach in terms of their personal qualities (e.g. as pleasant, caring, honest and practical). The role of the coach was described as being enabling, motivational, non-judgmental, giving encouragement and support. Patients valued the fact that they were in control and commented on the importance of guidance, in terms of advice giving, direction, and motivation.

... I wasn't sure if it would be her telling me to do this exercise and that exercise, but it's not like that at all. So it's empowering because when you have depression you feel like you've got no power over anything, so for her to say you're in charge of getting better was brilliant. (Patient 9)

### Patients' experience of outcomes and anticipated future use of guided self-help

Most patients selected behavioural activation as their preferred intervention (the other options were cognitive restructuring and ways to improve physical problems such as sleeping, irritability and concentration – see Table 5). They were attracted by the diary keeping, activity planning and goal setting. Patients described themselves as feeling much better as a result of their experience of guided self-help, having more confidence for the future and the knowledge that they can continue to use the techniques now or in the future.

[I'm feeling] tons better....I still have difficulty with some things because it doesn't clear overnight. (Patient 1)

### Phase 2 Effectiveness

The main analysis showed no significant benefit associated with the intervention on either primary or secondary outcomes (Table 10). However, as the sample size was small, the statistical power was low and the confidence intervals were wide. Effect sizes (i.e. mean difference divided by the pooled standard deviation) were calculated for primary and secondary outcomes to give an estimate of the magnitude of differences between groups and to allow comparisons with other published self-help studies.

**Table 10:** Clinical outcome at 3 month follow-up

**Outcome**	**Guided self-help** **(M, SD, n)**	**Usual Care** **(M, SD, n)**	**Adjusted mean differences (95% CI)**	**P value for group effect**	**Effect size**	**Adjusted effect size**
**BDI (II)**	18.74 (12.96) (19)	22.26 (12.40) (23)	-1.98 (-8.99 to 5.02)	0.57	-0.28	-0.18
**CORE-OM**	13.47 (8.33) (19)	13.61 (8.29) (23)	0.62 (-4.21 to 5.45)	0.80	-0.01	0.08
**SAS**	2.45 (0.61) (19)	2.59 (0.55) (21)	-0.05 (-0.38 to 0.28)	0.77	-0.24	-0.10
**PHQ9**	10.21 (7.30) (19)	10.81 (5.80) (21)	-0.05 (-4.10 to 3.99)	0.98	-0.09	-0.01

M – mean; SD – Standard deviation; n – sample size. See text for details of outcome measures (BDI; CORE-OM; SAS; PHQ9)

Three of the four effect sizes based on the unadjusted data showed an advantage for guided self-help which was 'small' by conventional standards. Because the small sample size meant that the groups may not have been well balanced at baseline, effect sizes were also calculated based on figures adjusted for baseline variables. In all cases, the adjusted effect sizes were smaller than the unadjusted figures.

**Figure 4:**
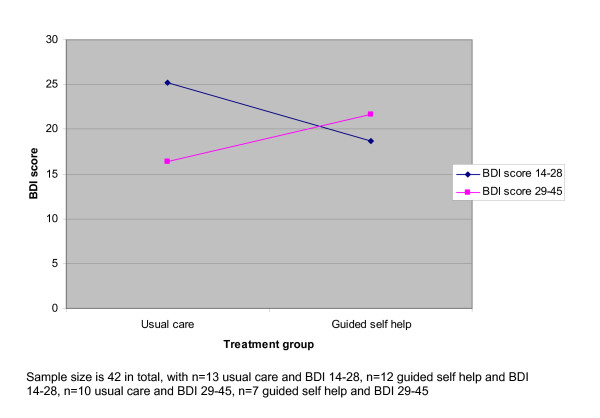
Relationship between treatment and predicted outcome in patients at different levels of baseline severity.

We conducted a subgroup analysis to examine the effect of initial severity on outcome, because of the change in recruitment inclusion criteria. This suggested severity had an effect which approached statistical significance (B = 11.830, standard error 6.684, p = 0.085). Figure 4 shows this interaction graphically. The plot shows the estimated regression slope between BDI outcome scores and treatment group. In the analysis without the interaction term, the slope would show a slight downward trend, indicating that moving from usual care to guided self-help is associated with a slight (but non-significant) reduction in BDI scores. In the plot which includes the interaction, separate slopes are plotted for patients in the 14–28 and 29–45 BDI baseline score categories. The slopes show that in patients with mild to moderate severity, moving from usual care to guided self-help is associated with a reduction in scores, whereas in patients with more severe problems, the opposite relationship is found. The statistical test indicates that the difference in the slopes (i.e. the difference in the effects of guided self-help in the two severity groups) approaches significance. Again, the limited sample size (n = 42, with cell sizes of 13, 12, 10 and 7) means that this result must be treated with caution.

## Discussion

The study has outlined an approach to the modelling phase of the Medical Research Council complex interventions framework, and provided some empirical data concerning the effectiveness of that modelling phase in the development of a specific intervention. The discussion will first consider the advantages and disadvantages of the general approach to modelling, and then move onto the interpretation of the data in the specific instance of the guided self-help intervention.

### Advantages and disadvantages of the approach to the modelling phase

As noted in the introduction, there is little consensus on the optimal methods to use in the modelling of complex interventions, and the present paper outlines one general approach which may have utility. The general principles of the modelling phase adopted in this study are designed to draw on a range of evidence. The meta regression synthesises best evidence from outcome studies, thus meeting the requirements for the intervention to be 'evidence based'. The meta synthesis draws on evidence from patients, assisting in the development of an intervention that is 'patient-centred'. The consensus process functions to draw these two forms of evidence together and deal with remaining ambiguities in ways that are transparent and acceptable to professionals and patients.

One of the methods adopted by this study (meta regression) has been used in previous complex intervention development work [23] while the other (meta synthesis) represents an innovation in this context. These methods have the advantage in that they are a relatively efficient use of research resources as they require no primary data collection.

Both methods provided useful information that was instrumental in developing the guided self-help intervention. The meta regression identified some aspects of delivery that were not associated with outcomes; this in turn enabled the guided self-help intervention to be delivered flexibly to meet patient needs without compromising effectiveness. The meta synthesis identified several core components of delivery, such as the importance of patient control and ensuring the patient is seen as the agent of change. The description provided by professionals and patients regarding acceptability suggested that the intervention was likely to coalesce with key elements of current practice and past experiences suggesting that integration and workability of the intervention and therefore its normalisation is likely to be successful. The consensus process was able to help overcome the gaps and ambiguities in the available evidence.

However, all the methods used in the modelling phase have disadvantages. The meta regression did not provide many major insights (for example, the finding that guidance and the use of CBT were associated with greater effectiveness simply repeated current consensus). Furthermore, meta regression has some significant methodological issues (e.g. sample sizes and power are generally limited, and the relationships between intervention factors and outcomes may not be causal) [23]. Other designs, such as dismantling studies [24], may be more effective in the modelling phase, albeit at significantly increased cost. Disadvantages of meta synthesis in the modelling phase include the fact that previously published studies may not provide very detailed data on the specific intervention under test. Where innovative interventions are being modelled, primary qualitative work may be required [25]. Some results of the meta synthesis (e.g. the importance of ensuring that the timing of the introduction of self-help is appropriate, given the patient's 'illness trajectory') proved difficult to introduce because of the way patients are routinely identified and referred within current services. Also, if insufficient quantitative and qualitative data about the intervention under test have been published to provide informative reviews, modelling may be very dependent on the consensus process, which may not be the most reliable tool. Because of limitations in funding and time, only a rudimentary consensus process was possible, without feedback of responses in multiple rounds. A more comprehensive process may prove useful in future studies.

### The exploratory trial of guided self-help

The next section will consider the results of the application of the modelling to the specific example of guided self-help for depression.

### Phase 2 Fidelity

In Session 1 the facilitators adhered to the manual and carried out the majority of the specific components. A notable exception related to *seeking feedback *(i.e. whether the therapist sought feedback from the client on the session itself and whether it was useful). This may reflect ambiguity in the rating manual, or that self-help coaches were being sufficiently vague in carrying out this component such as to make it difficult to rate reliably. In terms of specific techniques, some procedures were used considerably less than others (e.g. modifying factors). For sessions 5 onwards, the quality of the data was constrained and did not provide a representative data sample and so drawing any conclusions from these is problematic. However, observations suggest that fidelity is less strong in subsequent sessions and again data suggested that *feedback *about the usefulness of the interventions within the guide was poorly adhered to. Kappas are vulnerable to multiple influences and require uniform recognition by raters in order to yield unity agreement. Categories which are more abstract are likely to prove more difficult to yield good agreement and this effect will be exacerbated where samples sizes are small and slight variation in delivery occurs as a function of therapists being responsive to clients requirements. Such a phenomenon may well be more likely to occur as therapy progresses.

It should be noted that there was some evidence that the correct 'dose' of guided self-help was not delivered. Although the analysis of fidelity showed that the content and process of the sessions that were delivered were in accordance with the protocol, it should be noted that the *range *of session numbers described in the protocol was 3–10, but the mean number of sessions actually delivered was 3.5 (SD 3.3) with 12 of 29 (41%) patients receiving less than the recommended 3 sessions.

### Phase 2 Acceptability

Acceptability to both self-help coaches delivering the intervention and patients receiving the intervention was high. The written materials were positively appraised by both patients and coaches, particularly the use of the 'recovery stories'. Patients generally characterised guided self-help as being about taking back control of their lives, of it being empowering and giving them choices of which approach to take.

### Phase 2 Effectiveness

No pilot study can provide a precise estimate of therapeutic effect, and the recruitment problems in the current trial were compounded by a relatively high level of attrition at 3 months. This meant that the sample size was small and the confidence intervals around the estimates wide. It is plausible that the treatment is having a large beneficial or negative effect, and a more precise estimate will require a much larger definitive trial.

Given the extensive development work that went into the intervention, and the relatively high levels of fidelity and acceptability, it was somewhat disappointing that the intervention could not achieve significant benefits for patients. The effect sizes were in line with previous analyses of guided self-help interventions in the United Kingdom [26-28]. Nevertheless it is noteworthy that the assumed benefits of the complex intervention development process and the extensive modelling phase have not translated into obvious benefits in effectiveness above those found in previous trials which have not been through such a comprehensive development procedure [26-28].

It is possible that guided self-help cannot function as a default intervention for the majority of patients with mild to moderate depression, and needs to be more effectively targeted. At present, the stepped care model which underlines the National Institute for Health and Clinical Excellence (NICE) guidelines for depression may be making unrealistic assumptions about the proportion of patients who can benefit from guided self-help, and adoption of a stratified model (with greater attention to identifying patients who are likely to respond to these treatments, and those who require alternatives) may be more effective. However, this requires identification of effective predictors of response, which are so far lacking.

There was some evidence from the subgroup analysis that treatment effect was moderated by initial severity, with patients who had mild to moderate problems showing greater benefit in guided self-help than usual care. It is possible that the change in inclusion criteria and subsequent recruitment of patients with more severe problems meant that the trial underestimated the effectiveness of guided self-help among patients with mild to moderate problems. However, two caveats apply. First, the small sample size means that precision of the estimate of the interaction is limited, and the results would require replication. Secondly, the reason for the change in inclusion criteria was that a relatively small proportion of patients demonstrated the mild to moderate level of severity, which would limit the potential utility of guided self-help in a stepped care system. The modest effectiveness of the intervention reported in this study does suggest that more effective targeting of the intervention may be required, and research looking at predictors or moderators of treatment effect should be a priority for the future [29].

### Limitations of the exploratory trial

Although most professionals provided tapes for the analyses of fidelity, these analyses were hampered somewhat by the restricted number and distribution of tapes available. The analysis of fidelity did not examine the presence of proscribed components (i.e. components which would be inconsistent with the procedure) or of common factors (i.e. components which would be deemed common to most psychological interventions). In addition, the competence with which the components were delivered was not rated. A facilitator may be rated highly on fidelity but low on competence.

The samples of patients and professionals interviewed for the analysis of acceptability were relatively small, and may have been biased. Professionals agreeing to take part in a trial may represent those who are open to new techniques, and acceptability may be lower in the wider professional population. Sustainability of the intervention (i.e. the degree to which it is used after the study) is a key issue in the complex interventions framework Phase 4 (see Figure 1), although no data was collected on this issue in the current study. Similarly, patients who agree to take part in a trial and in a qualitative interview to discuss the treatment may not be representative of the wider population to which guided self-help might be offered. It is noteworthy that all but one of the 9 patients interviewed had received more than 1 session. Patients who fail to engage with treatment are likely to be those who are also difficult to recruit to further research such as qualitative interviews.

The study failed to meet the recruitment target. Recruitment to primary care trials in the United Kingdom is routinely problematic [30,31]. Different challenges were faced in each site. In one site, recruitment was low due to the high proportion of patients whose severity of depression rendered them ineligible (the proportion with severe problems was significantly higher than estimated). This led to a change in the eligibility criteria, to improve recruitment and better reflect the sorts of patients currently receiving this treatment in routine treatment. In the second site, recruitment foundered because patients were being seen for routine treatment too rapidly, which meant that patients offered the trial were being asked to delay accessing conventional treatment and risk being allocated to a usual care group for 3 months.

The United Kingdom has seen the introduction of new organisations and resources to assist with recruitment to trials [32]. Although these may aid recruitment, some of the key barriers to the recruitment of depressed patients in primary relate to issues such as the low motivation of depressed patients to engage in extra activities, concerns among professionals about the inclusion of vulnerable patients into research studies and anxieties about randomisation to treatment [31], and the difficulties of introducing research into contexts where patients are upset [33]. The ability of networks to overcome these barriers remains an important empirical question for the future.

## Conclusion

The study used an intervention development process that enabled the production of an intervention that was in line with current best evidence, acceptable to patients and professionals and amenable to delivery in primary care. However, the development process resulted in an intervention that did not markedly improve outcomes in an exploratory trial.

## Competing interests

The authors declare that they have no competing interests

## Authors' contributions

KL, PB, CR, DR, MB, BS, LD, AR conceived, designed and conducted aspects of the study, obtained funding and drafted the manuscript. JG and SH helped conduct the study and contributed to analysis. CR and PB conducted the statistical analysis. All authors contributed to the interpretation of the results and the final manuscript.

## Pre-publication history

The pre-publication history for this paper can be accessed here:


